# A comparison of several separation processes for eggshell membrane powder as a natural biomaterial for skin regeneration

**DOI:** 10.1111/srt.70038

**Published:** 2024-09-10

**Authors:** Samaneh Esmaeili, Majid Rahmati, Sepehr Zamani, Ali R. Djalilian, Zohreh Arabpour, Majid Salehi

**Affiliations:** ^1^ Student Research Committee School of Medicine Shahroud University of Medical Sciences Shahroud Iran; ^2^ Department of Medical Biotechnology School of Medicine Shahroud University of Medical Sciences Shahroud Iran; ^3^ Department of Ophthalmology and Visual Sciences University of Illinois at Chicago Chicago Illinois USA; ^4^ Tissue Engineering and Stem Cells Research Center Shahroud University of Medical Sciences Shahroud Iran; ^5^ Department of Tissue Engineering School of Medicine Shahroud University of Medical Sciences Shahroud Iran

**Keywords:** biocompatible materials, eggshell, extracellular matrix, migration, wound healing

## Abstract

**Background:**

Numerous studies have focused on skin damage, the most prevalent physical injury, aiming to improve wound healing. The exploration of biomaterials, specifically eggshell membranes (ESMs), is undertaken to accelerate the recovery of skin injuries. The membrane must be separated from the shell to make this biomaterial usable. Hence, this investigation aimed to identify more about the methods for membrane isolation and determine the most efficient one for usage as a biomaterial.

**Methods and materials:**

For this purpose, ESM was removed from eggs using different protocols (with sodium carbonate, acetic acid, HCl, calcium carbonate, and using forceps for separation). Consequently, we have examined the membranes' mechanical and morphological qualities.

**Results:**

According to the analysis of microscopic surface morphology, the membranes have appropriate porosity. MTT assay also revealed that the membranes have no cytotoxic effect on 3T3 cells. The results indicated that the ESM had acquired acceptable coagulation and was compatible with blood. Based on the obtained results, Provacol 4 (0.5‐mol HCl and neutralized with 0.1‐mol NaOH) was better than other methods of extraction and eggshell separation because it was more cell‐compatible and more compatible with blood.

**Conclusion:**

This study demonstrates that ESMs can be used as a suitable biomaterial in medical applications.

## 1 INTRODUCTION

The skin is the protective barrier against the external environment and invading germs. The skin's anatomical layers have been subdivided into four functional cutaneous barriers: physical, chemical, immunological, and microbial, each responsible for various protective activities.^1^ Skin damage can result from a variety of traumas, including burns, severe injury, infection, and cancer. Healing a wound is a complicated process involving several different cell types and cytokine mediators being activated at once.^2^ Furthermore, chronic wounds may occur due to diseases like peripheral vascular disease or diabetes mellitus, raising the risk of infection and other problems.^3^ During abnormal tissue regeneration, impaired angiogenesis and nutrition delivery lead to chronic conditions and the ongoing stimulation of inflammatory cells around the wound area. Hence, wounds are a crucial financial healthcare system burden and a physical, mental, and social problem for the person.^4^ Most superficial wounds heal on their own; on the other hand, full‐thickness wounds do not heal on their own and take a very long time to recover. It requires some specific biomaterials to fill the severely damaged area, which helps speed up the healing of the wound.^5^ The biomaterials for wound healing must be rich in biopolymers/ biomolecules, which can increase the rate of wound healing.^6^ Today, biomaterials play numerous roles in regenerative medicine, tissue engineering,  biosensors, biotemplates, and more^7^; recent advances in biomaterials have provided opportunities for clinical use. However, the principal challenge of using appropriate biomaterials for biomedical applications is the lack of biocompatibility, often related to allergies or inflammation reactions, blood homeostasis, tumorigenesis, altered viscoelasticity of extracellular matrix (ECM), biodegradability, and multiple drug resistance.^7^ Eggshell membrane (ESM) has been utilized as a material to accelerate wound healing.^8^ ESM is a biopolymeric membrane that is one of the most abundant industrial bio‐waste materials; this high‐value waste can benefit medical applications.^9^ ESM is the bilayer, a noncalcify membrane between the shell and albumen. The ESM has a fibrous meshwork of two thin submembranes: an outer membrane that is thicker and linked to the shell and an inner membrane that is thinner and attached to the egg white.^10^ The ESM comprises bioactive compounds, including hyaluronic acids, chondroitin sulfate, dermatan sulfate, and glycoproteins. This structure is also mainly formed by proteins (∼90%), and proteomic examination of it has demonstrated about 500 proteins, containing glycoproteins, elastin, sialoprotein, osteopontin, collagens (I, V, and X), as well as functional proteins like lysozyme and ovotransferrin.^11^, ^12^ In addition, the ESM has properties essential for wound healing, such as appropriate moisture retention, air permeability, and the ability to adhere to textured surfaces due to its network structure.^13^, ^14^ Also, ESM is similar to the natural ECM to some degree. Because of this, it is a unique candidate as a biomaterial source for the effective regeneration of tissue and joint pain and for preventing skin aging.^15^, ^16^ According to preliminary research, Yang et al. say that putting ESM directly on the location of skin grafts helped alleviate pain and protect the wound.^17^ Also, in the initial stages of wound recovery, Guarderas et al. and Jun et al. demonstrated that ESM enhances wound closure during regeneration.^18^, ^19^ Glycosaminoglycans (GAGs) in the ESM are intriguing candidates for wound healing mediators because of their anti‐inflammatory activities.^20^ Also, Some investigations have found that solubilized and hydrolyzed ESM has anti‐inflammatory effects.^17^ Mensah et al. in a study investigating the regenerative effects of the ESM in corneal wounds acknowledged that it has excellent biocompatibility with human epithelial cells (ihCEC) and corneal mesenchymal stromal cells (C‐MSC).^21^ In another study, Tram T. Vuong, by examining the function of eggshell in cell activity and the expression of MMP_2,9_ that play an important role in the initial phase of wound healing, stated that eggshell powder increases the expression of MMPs, and the constructive effect and positive effects on cell function can play an important role in wound healing.^22^ According to another research, ESM exhibits good biocompatibility. Hence, with the increasing use of ESM, it is essential to develop a separation technology to be used on a large industrial scale. Since using acid solutions to separate minerals from eggshells is common, it is crucial to learn more about these acids' impact on the membrane's chemical composition and biological properties. As a result, to benefit from this biopolymer, the entire membrane must be separated from the mineral shell. While many studies are employing various methods to isolate ESM, more information must be provided to compare the efficacy of different ESM extraction techniques.

This paper aims to determine which protocol is better for extracting this valuable biomaterial. To do this, we used six extraction methods to compare the biological characteristics of the natural ESM. Finally, the structural and morphological characteristics of ESM and its compatibility with blood and fibroblast cells were investigated in order to obtain a deeper insight into the optimal extraction method of ESM and to evaluate its performance when used for wound healing in laboratory conditions.

## 2 MATERIALS AND METHODS

### 2.1 Materials

Chicken eggs were bought from a nearby store. All reagents and organic solvents acetic acid (CH_3_COOH), acetone, hydrochloric acid (HCl), sodium hydroxide (NaOH), phosphate‐buffered saline (PBS), fetal bovine serum (FBS), Dulbecco's modified Eagle medium (DMEM‐F12), penicillin‐streptomycin solution (pen‐strep solution), 3‐(4, 5‐dimethylthiazol‐2‐yl2, 5‐diphenyltetrazolium bromide) (MTT), trypsin‐ethylene diamine tetra acetic (trypsin‐EDTA)were purchased from Merck and Sigma‐Aldrich.

### 2.2 Natural eggshell membrane powder preparation

First, the fresh eggs were washed with deionized (DI) water. Afterwards, a small hole was punctured at the tip of the eggs, and the contents were removed. Subsequently, the inside of the eggs was cleaned three times with DI water. Then the ESM was separated from the shell using the following protocols at room temperature.

Protocol 1 (P1): A 0.05‐M solution containing sodium carbonate was used to wash the eggshells. Following that, membranes were given a wash in a solution of distilled water. And the ESMs were separated from the eggshells.^23^

Protocol 2 (P2): After the eggs had been emptied, 5% (v/v) acetic acid was added to them, and they were left to remain for 45 min at 37°C. Next, the membranes were removed from the calcified portion of the eggshell with great care, and the following three times washed with acetone, and, in the end, the membrane was cleaned with distilled water.^24^

Protocol 3 (P3): When the eggs had been depleted, 24‐h immersion in a solution contains 1% acetic acid to remove any eggshell fragments that may still exist in the membranes. Afterwards, membranes were rinsed in DI water.^25^

Protocol 4 (P4): After the albumin had been extracted from the eggs, the emptied eggs were immersed in diluted 0.5‐mol HCl for 24 h to get rid of their ES. The membrane of the eggshell was extracted, and the ESM was neutralized with 0.1‐mol NaOH for 1 h. Next DI water was used to wash the membranes.^26^

Protocol 5 (P5): After removing the albumin from the eggs, the emptied eggs were soaked in 0.5‐M acetic acid for a total of 44 h; once the calcium carbonate shell had been dissolved completely, the extracted membranes were gathered and washed with DI water.^21^

Protocol 6 (P6): As a control group, after albumin had been extracted from eggs, the membranes of the eggshell were separated using forceps from the calcified eggshell and cleaned with DI water.

All collected ESM samples were freeze‐dried for 2 days and were subsequently pulverized using liquid nitrogen to a powder for a short time. The preparation powder was stored at 4°C.

### 2.3 Characterization of the ESM

#### 2.3.1 Analysis of microscopic surface morphology

The morphology of the surface of the ESM was observed under scanning electron microscopy (SEM; DSM 960A, Zeiss, Germany). Each sample was operated in different sizes after being sputter‐coated in gold. Imaging was done at a 15‐kV accelerating voltage, and software for image analysis was employed to find the average size of the pores.^27^

#### 2.3.2 Functional group identification

Functional groups of ESM were identified using a NICOLET‐6700 FTIR spectrophotometer (Thermo Scientific). The study was conducted in 400–4000 cm^−1^ to find the highest point of O, C, and N.^28^

### 2.4 Cytotoxicity assay

The ISO 10993‐5 standard was used to conduct the cytotoxicity test. The MTT analysis was conducted to assess the viability and cytotoxicity of the manufactured ESM. Before cell seeding, ESM was placed for sterility under UV radiation for 20 min. The 3T3‐ murine fibroblast cell line used in this study and about 1 ×10^4^ cells were seeded into ESM in a 96‐well plate, which involved Dulbecco's modified Eagle's medium (DMEM) with 10% FBS, and 1% (v/v) pen‐strep solution. The incubator's atmosphere was 37°C and 5% carbon dioxide, and the culture plates were put inside. At 24 and 72 h after cell seeding, 100‐µL MTT reagent was applied for every well at a final concentration of 0.5 mg mL^−1^. Afterwards, the plates were incubated for another 4 h at 37°C in an incubator to determine cell viability. Following the removal of the medium, 100 µL of dimethyl sulfoxide (DMSO) solution was put in every well. The optical density of each well at 570 nm was measured with a 96‐well microplate reader. The wells in a tissue culture plate with no cells were called “blank,” while the wells with cells and without ESM were called “control.” Using the following formula, the proportion of viable cells in every sample was calculated.^29^

$$\mathrm{Cell}\mathrm{viability}\%\;=\;\frac{\mathrm{Sample}\;\mathrm{absorbance}\;\mathrm{value}}{\mathrm{Control}\;\mathrm{absorbance}}\;\times \;100$$

### 2.5 Wound scratch assay

A wound‐healing assay was carried out by scratching the confluent culture to assess the migration of 3T3 cells. At a density of 5 ×10^3^ 3T3, cells on a six‐well plate were seeded. The medium was changed every 2 days, and culture was kept up until about 80% confluence. The medium was removed, and a scratch was formed with a micropipette tip. After that, the cultures were fed with the recommended doses of ESM. As a control, cells with no supplement were employed. An inverted microscope was used to take pictures of the wound site on day 0 (the day after the scratch) and day 2. The migration process was captured in digital images using a computer‐connected microscope and Image J software (National Institutes of Health, Bethesda, USA).^30^

### 2.6 ESM measurements of moisture content and drying

Determining the amount of moisture in a product requires weighing it, drying it thoroughly, and reweighing it. So for this study, ESM was extracted from eggs, and then the ESM was placed on a plate and weighed. After drying with a freezer dryer for 48 h, the dried ESM were placed on a preweighed plate and reweighed.^31^ The moisture content of ESM was estimated utilizing the following formula:

$$\mathrm{Moisture}\mathrm{content}\left(\%\right)\;=\;\frac{\mathrm{Ww}-\mathrm{Wd}}{\mathrm{Ww}}\;\times \;100$$

where *W*_d_ = weight of the ESM after drying and *W*_w_ = wet weight of the ESM.

### 2.7 In vitro hemocompatibility assays

In this experiment, 2.5 mL of normal saline was used to dilute 2 mL of fresh anticoagulated human blood. After that, a blood‐dilution solution (0.2 mL) was added to the ESM vials. The samples were maintained at a temperature of 37°C for 1 h, before being centrifuged at a rate of 1500 rpm for 10 min. The supernatants that were made were put in a 96‐well plate, and the absorbance at 545 nm was measured with a Microplate Reader. The average of the three readings was determined. Negative controls included 10 mL of normal saline and 0.2 mL of dilute blood, whereas positive controls included 0.2 mL of diluted blood in 10 mL of DI water. The degree of hemolysis was computed as follows.^32^

$$\mathrm{Hemolysis}\left(\%\right)\;=\;\frac{Ds-D\mathrm{nc}}{D\mathrm{pc}-D\mathrm{nc}}\;\times \;100$$

where *D*_nc_ represents the negative control absorbance, *D*_s_ represents the sample's absorbance, and (*D*_pc_) the absorbance of the positive control.

### 2.8 Blood coagulation index (BCI)

Samples (the produced ESMs powders) weighing 10 mg were put into a beaker in a thermostatic water bath, and100‐µL anticoagulated human blood was dumped on them. Next, all specimens were placed at 37°C for 5 min. In addition, 20 µL of a 0.2‐mol/L CaCl_2_ solution were transferred to samples. Five minutes later, 25 mL of distilled water was added. At 37°C, the sample was well agitated, also the absorbance of solutions was determined at 545 nm. There were no samples in the control group, and the average of three samples from each group was measured simultaneously.^33^

$$BCI\;=\;\frac{A\mathrm{sample}}{A\mathrm{control}}\;\times \;100\%$$

Each experimental sample has an absorbance value, denoted by *A*_sample_, and in the absence of the sample, the control group's absorbance is denoted by *A*_control_.

### 2.9 In vitro degradation study

The dried ESMs were placed in a test tube with 5 mL of PBS and separately in trypsin‐EDTA liquid for in vitro degradation research. The tubes were maintained for 30 days while kept sterile and at 37°C. After that, samples were taken, cleaned with purified water, and then dried. In addition, every sample was also weighed, and the degradation rate was determined using the equation below.^34^

$$\mathrm{Degradation}\;\%\;\;=\;\frac{W0-Wt}{W0}\;\times \;100$$

where *W*_0_ is the primal sample weight and *W*_t_ represents the weight of the deteriorated samples at various times.

### 2.10 Microenvironmental pH

Fifty milligrams of ESMs was cut and then submerged in 2 mL of 0.9% normal saline solution for 48 h at room temperature. The pH of the immersion solution was analyzed to recognize the microenvironment's pH of the ESM.^35^

### 2.11 Statistical analysis

The information was analyzed with the GraphPad Prism application. Each experiment was repeated three times, and the results are shown as the mean SD. To compare the groups, one‐factor analysis of variance (ANOVA) with the Tukey's post‐hoc test. Data were compared using the Kruskal–Wallis H nonparametric ANOVA test and then the Mann–Whitney *U* test when significant. The *p*‐value was taken into account and statistical significance was assigned to the value *p* < 0.05.

## 3 RESULTS

### 3.1 Morphological analysis

**FIGURE 1:**
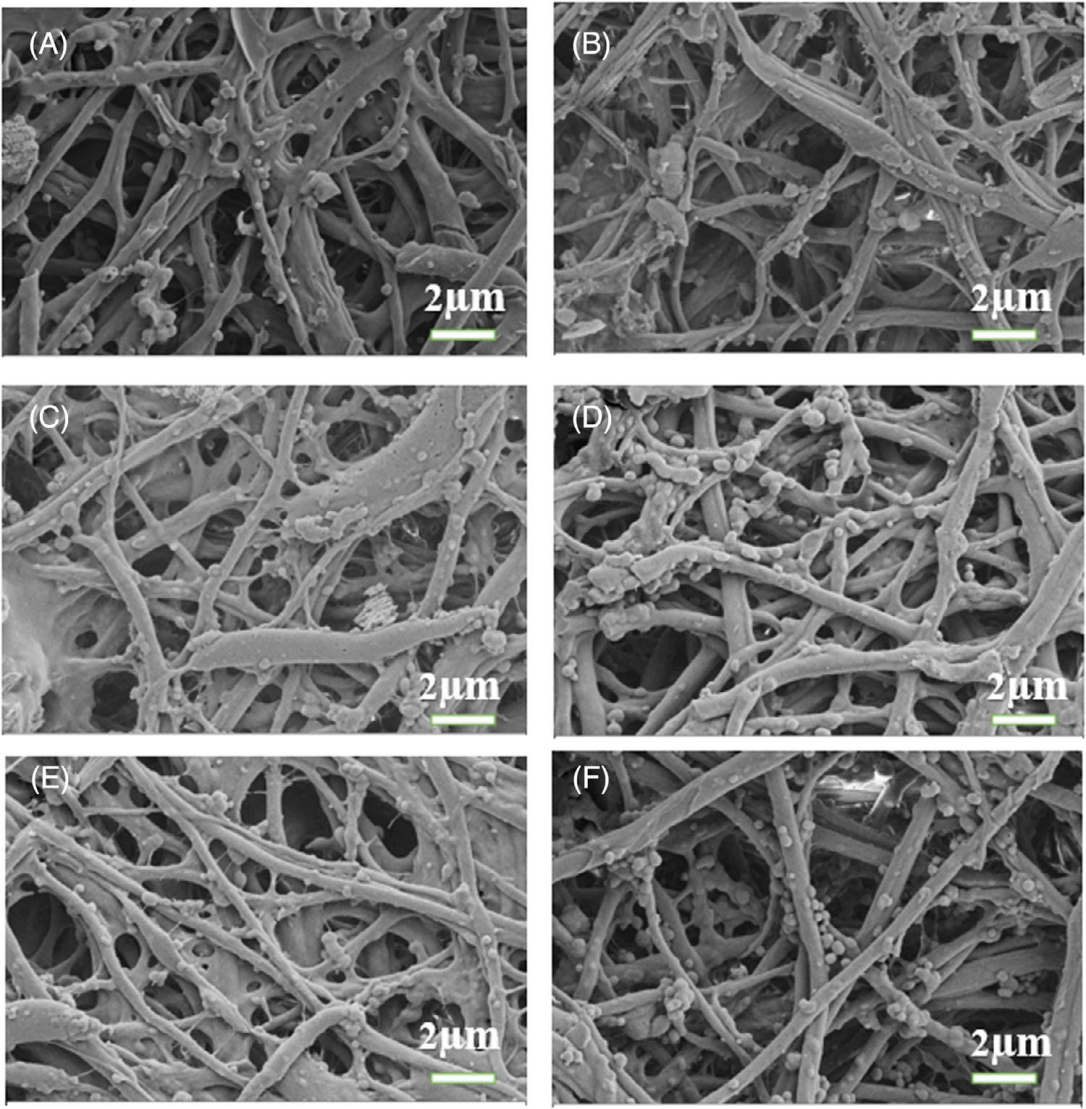
Morphology of the eggshell membrane. (A) P1, (B) P2, (C) P3, (D) P4, (E) P5, and (F) P6. Magnification: 5.00 K.

The morphological properties of ESM were recognized by SEM micrographs (Figure 1). Images demonstrated that the morphology of the fibers was randomly aligned. SEM analysis shows most of the ESM as dense clusters of strong thread‐like fibrils due to its intrinsic similarity to the ECM. The spherical structures show the cracks caused by the extraction process in the structure. In addition, the obtained images are similar to other studies.^36^ Using ImageJ software (NIH, Bethesda, USA), all groups' average fibril diameter was statistically estimated. The membrane's pore morphology, size, interconnectivity of pores, and porosity are crucial characteristics that directly impact cell proliferation. The examination of the pictures revealed that the average size of pores in the ESM was approximately 416.5 nm to 6.952 µm. The resulting pore size is appropriate for cell invasion and migration. These data demonstrated that the produced ESM is suitable for tissue regeneration applications, which demand a porous structure with interconnected pores and enough pore size.

### 3.2 Functional groups identification

FTIR‐KBr analyses demonstrated the change of the related absorption peaks in the ESMs' organic structure, as demonstrated in Figure 2. ESMs obtained through different methods of separation yielded FTIR‐KBr spectra that varied in the distinctive absorption peaks of the organic contents and also in absorption bands associated with the ESM. The protein/amide signature bands showed three extremely strong bands. A wide band in the 3400–3500 cm^−1^ frequency range (amide A: stretching vibration of the NH group) as well as more defined bands in 1600–1670 cm^−1^ (amide I: stretching vibration of the C = O group) peak at 1530–1540 cm^−1^ (amide II: NH in‐plane bending and CN stretching).^15^, ^37^ Also, 1234 cm^−1^ (NH in‐plane bending and CN stretching). Amide vibrations of the fiber glycoprotein can be attributed to these regions.^38^ The spectra of ESMs that were manually separated and those that were separated using an acid dissolution of the membranes exhibited variances in the existence of absorption bands and differences in the shape and intensity of those bands. The membrane spectra acquired with HCl treatment showed the organic components' typical absorption bands of the ESM identical to the FTIR spectra produced manually by ESM separation (Figure 2c,e).

FIGURE 2FTIR spectra for the eggshell membrane. (A) P1, (B) P2, (C) P3, (D) P4, (E) P5, and (F) P6.

**Figure:**
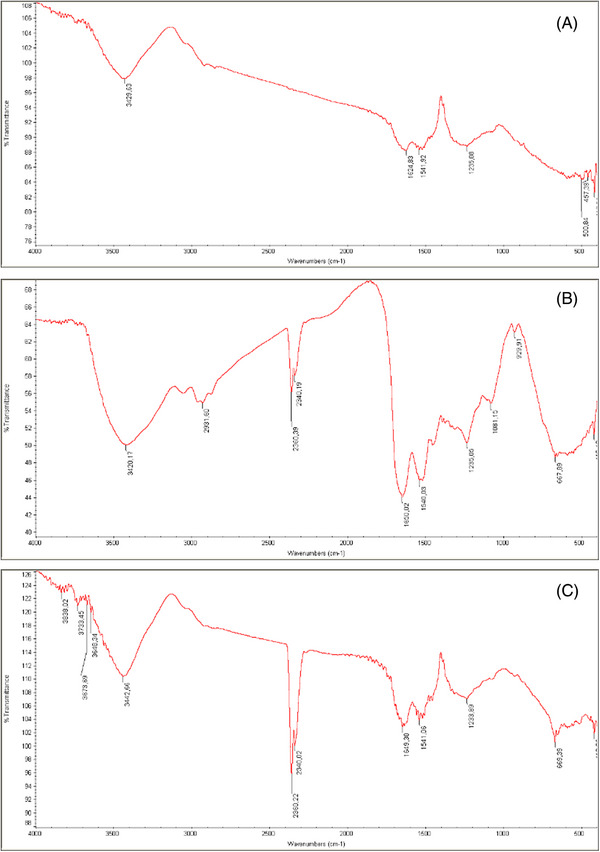

**Figure:**
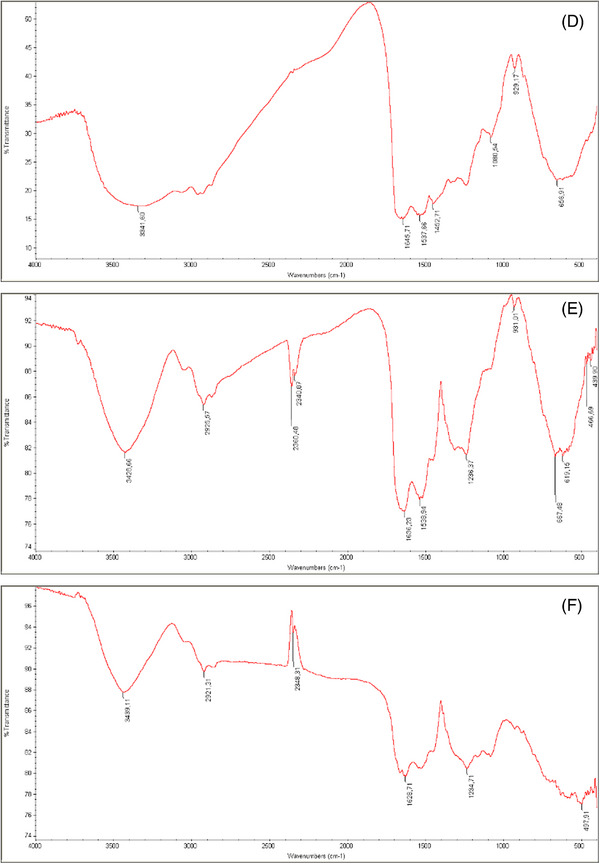

### 3.3 Findings on cell viability

**FIGURE 3:**
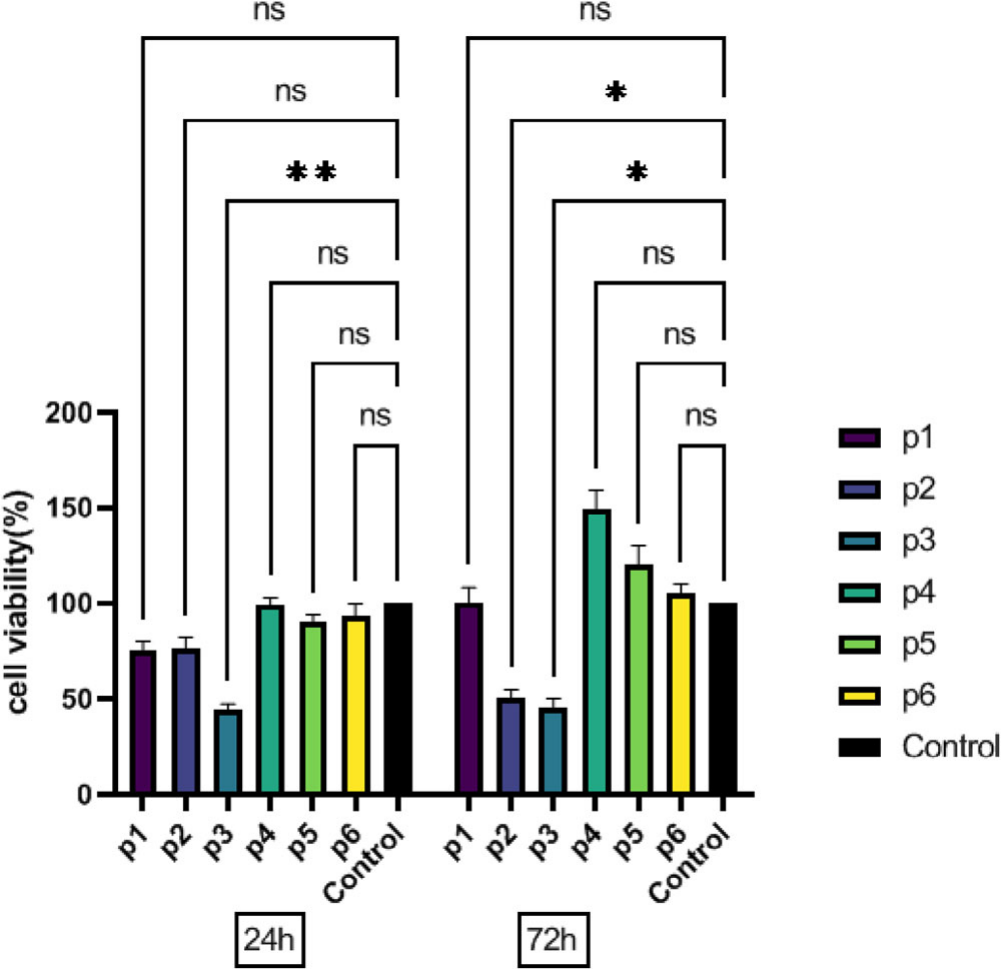
MTT assay of eggshell membrane after 24 and 72 h of 3T3 culture. Values represent the mean ± SD, *n* = 3. Significance levels are expressed as: ****p < .0001, ***p < .001, **p < .01, and *p < .05.

The cytocompatibility of the ESM was tested using an MTT experiment, and the findings were displayed in (Figure 3). After 24 and 72 h, P4 had higher cell viability values than the other groups. Moreover, the rate of cell proliferation was significantly greater than that of the control group. However, the viability of cells in P3 was considerably reduced 72 h after cell seeding. The results demonstrated that the manufactured ESM was cytocompatible and promoted the growth of 3T3 cells.

### 3.4 Wound healing assay

**FIGURE 4:**
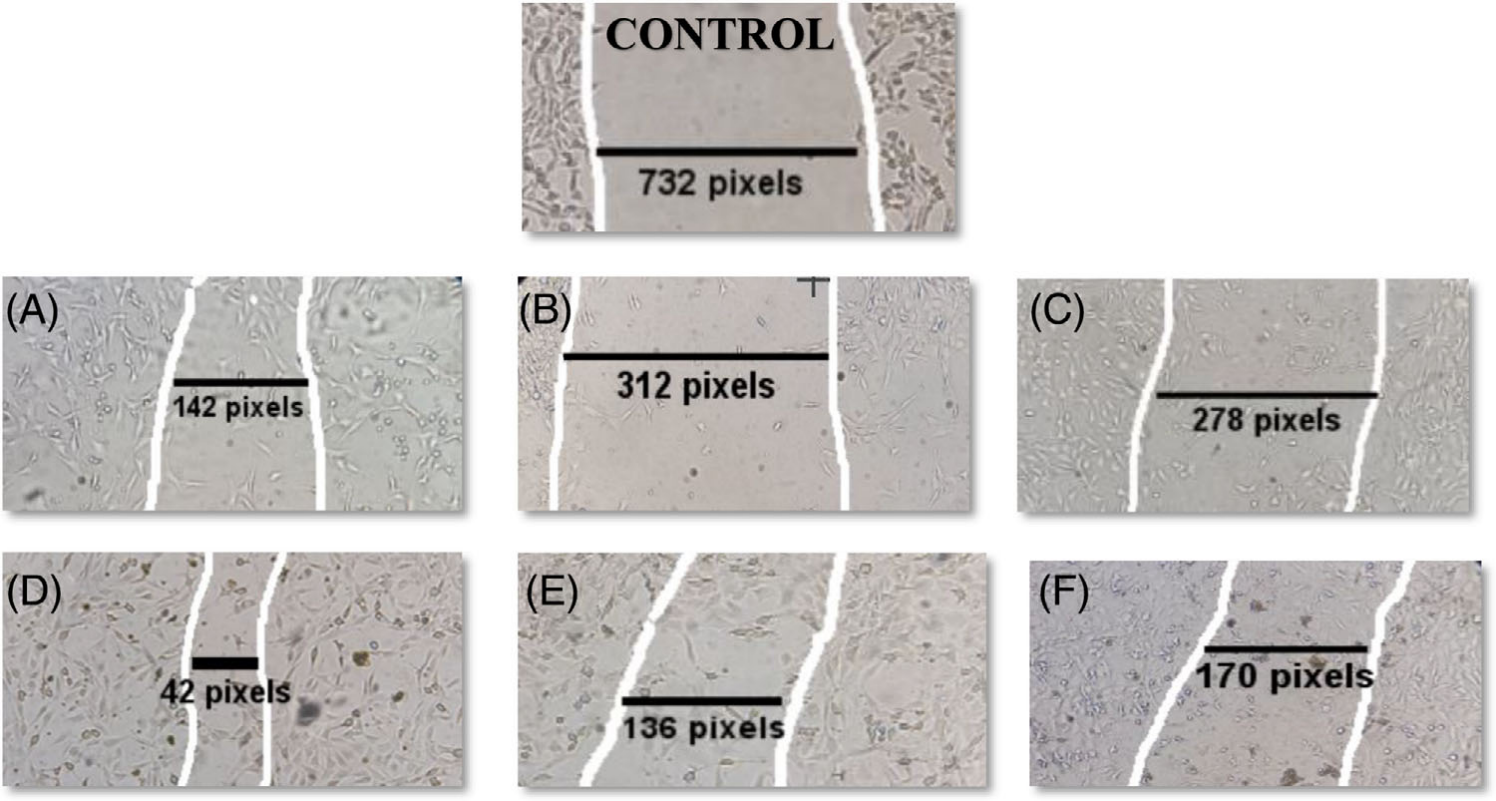
Representative pictures of 3T3 cells grown in control and test conditions healing wounds in vitro (supplemented with eggshell membrane conditions). The lines represent the scratch width. (A) P1, (B) P2, (C) P3, (D) P4, (E) P5, and (F) P6.

**FIGURE 5:**
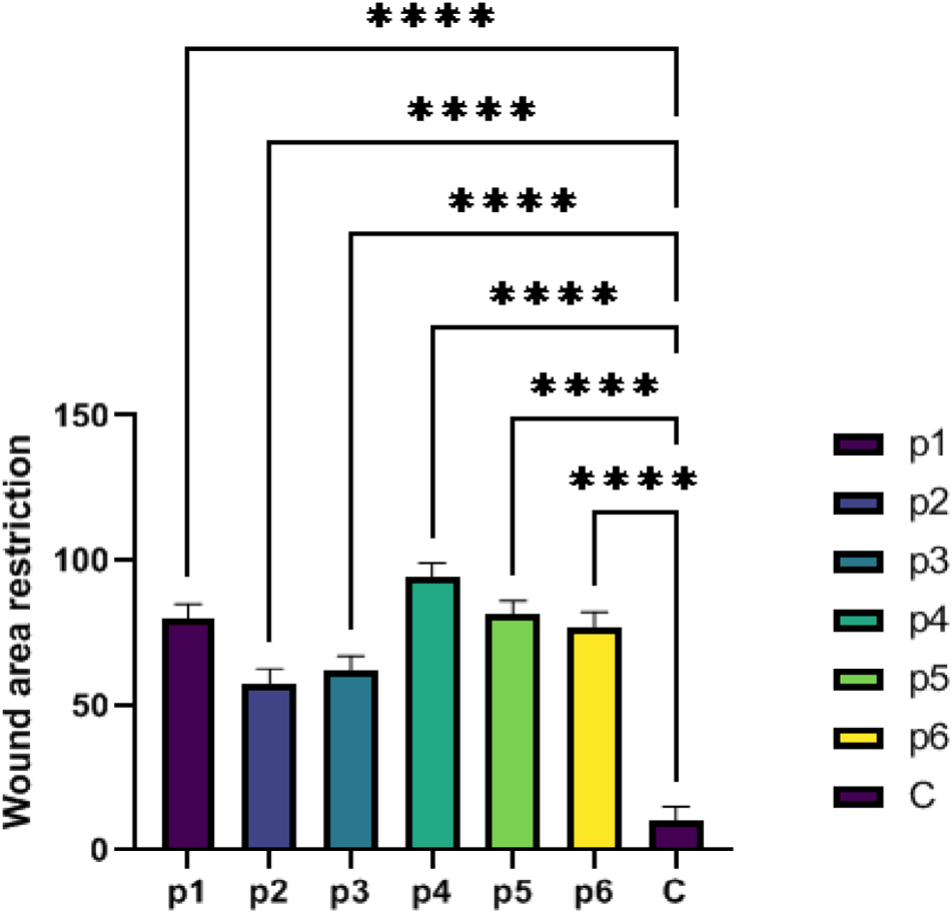
Migration rate of 3T3 cells cultured supplemented with the eggshell membrane in test conditions and a control group. Image J software calculated the migration rate by measuring the distance traveled by cells. Values represent the mean ± SD, **p < .01 and ***p < 0.001. ns., not significant; SD, standard deviation.

The scratch wound assay was applied to evaluate migration of 3T3 cells in vitro in confluent culture. The rate of cell migration was assessed 2 days after wound creation in 3T3 cell cultures supplemented with ESMs and a control group. The sample images of the migration of 3T3 cells on days 0 and 2 following wounding are shown in Figure 4 in control cultures and cultures supplemented with ESM. The culture supplemented with ESM P4 3T3 cells migrated more quickly, and the wound area recovered substantially faster than in the other groups within 2 days. Compared to control cultures, the migration rate of 3T3 cells in the culture treated with ESM was considerably higher (Figure 5). However, in P2, the rate of migration of 3T3 cells decreased significantly and was the same as in the control culture. These findings show that ESM considerably affects the migration rate of 3T3 cells in vitro, which will benefit wound healing re‐epithelization.

### 3.5 Moisture content

**FIGURE 6:**
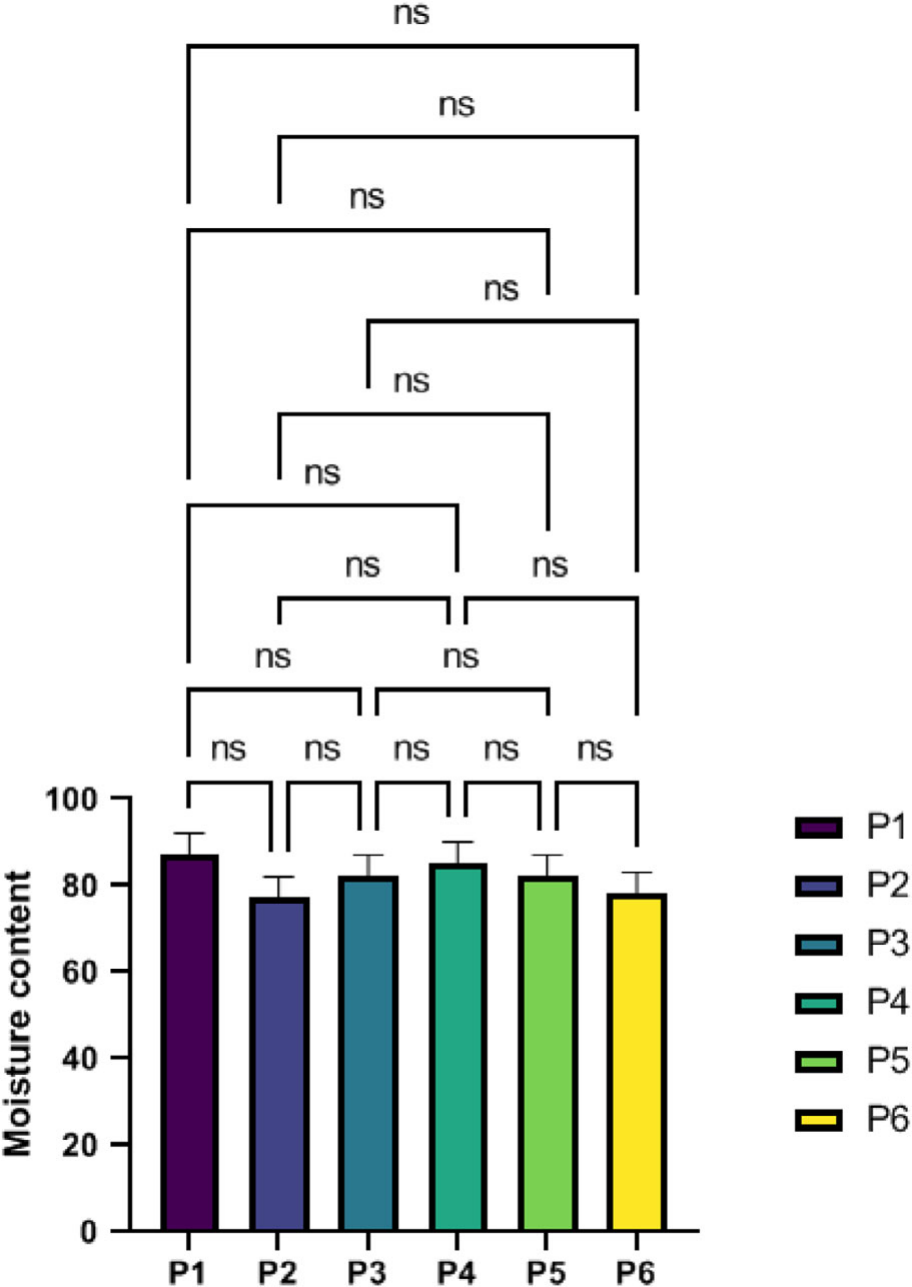
Results of eggshell membrane moisture content measurements values represent the mean ± SD, *n* = 3. Significance levels are expressed as ****p < .0001, ***p < .001, **p < .01, and *p < .05.

Figure 6 displays the ESMs' moisture content. The moisture content of the ESM was identified by drying samples in a freezer dryer until they attained a consistent weight. The maximum moisture content was reported for P1, whereas the lowest was for P2. However, all of the samples had a similar level of moisture content.

### 3.6 Blood compatibility

**FIGURE 7:**
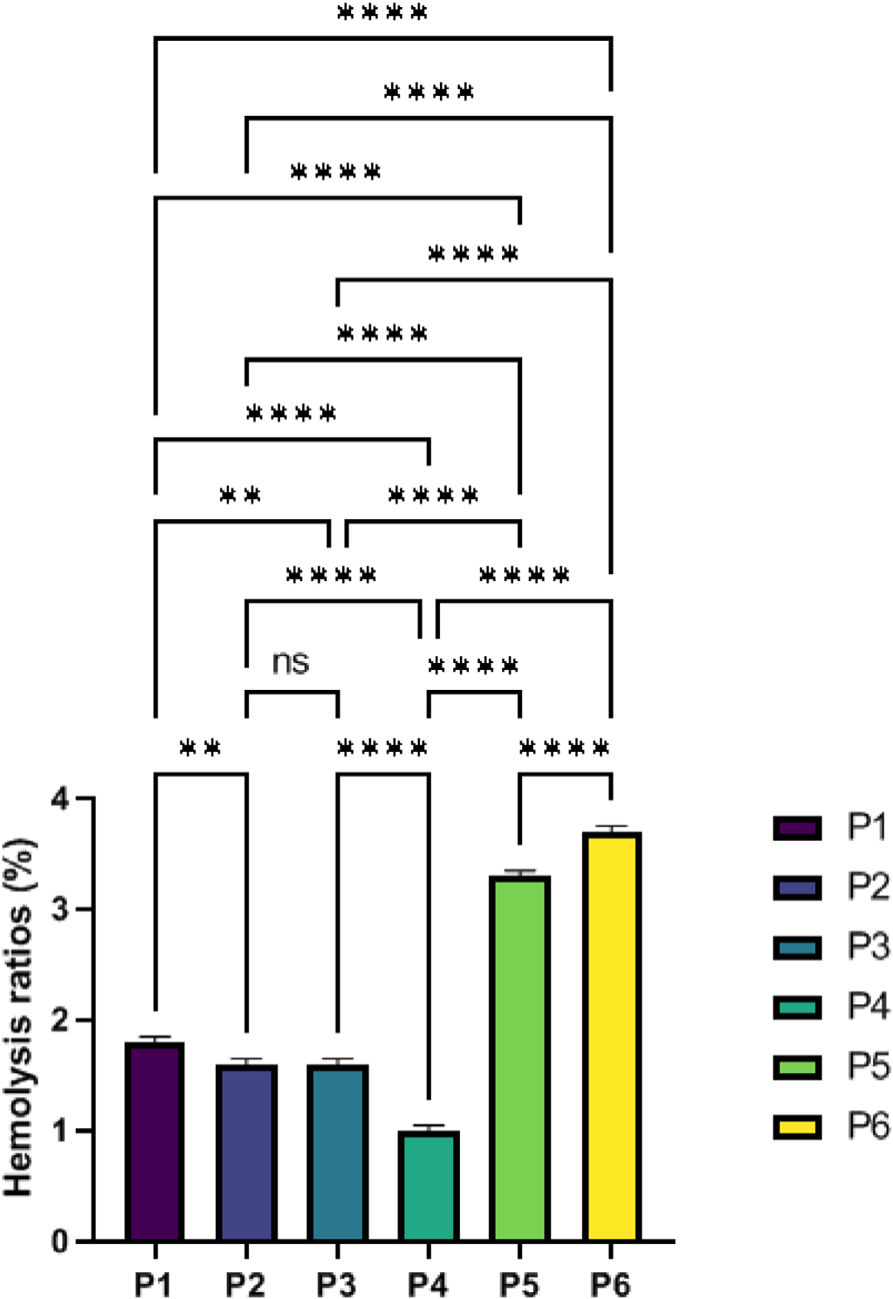
The hemocompatibility assay of eggshell membrane. Values represent the mean ± SD, *n* = 3. Significance levels are expressed as ****p < .0001, ***p < .001, **p < .01, and *p < .05.

Blood compatibility of biomaterials is directly related to hemolysis, which reveals the release of hemoglobin into plasma as a result of erythrocyte destruction. One of the significant aspects in the successful treatment of wounds is the produced wound dresser's compatibility with cellular components of the blood, particularly erythrocytes.^39^ The contact between the wound dresser and erythrocytes in the first phase after implantation ultimately decides the secondary bodily reactions, like inflammation. As in Figure 7, overall, hemolysis rates on all ESMs were lower than the positive control, and the difference was statistically significant. This showed that the membranes were very compatible with blood, which means they can be used in regenerative medicine.

### 3.7 Blood coagulation index (BCI)

**FIGURE 8:**
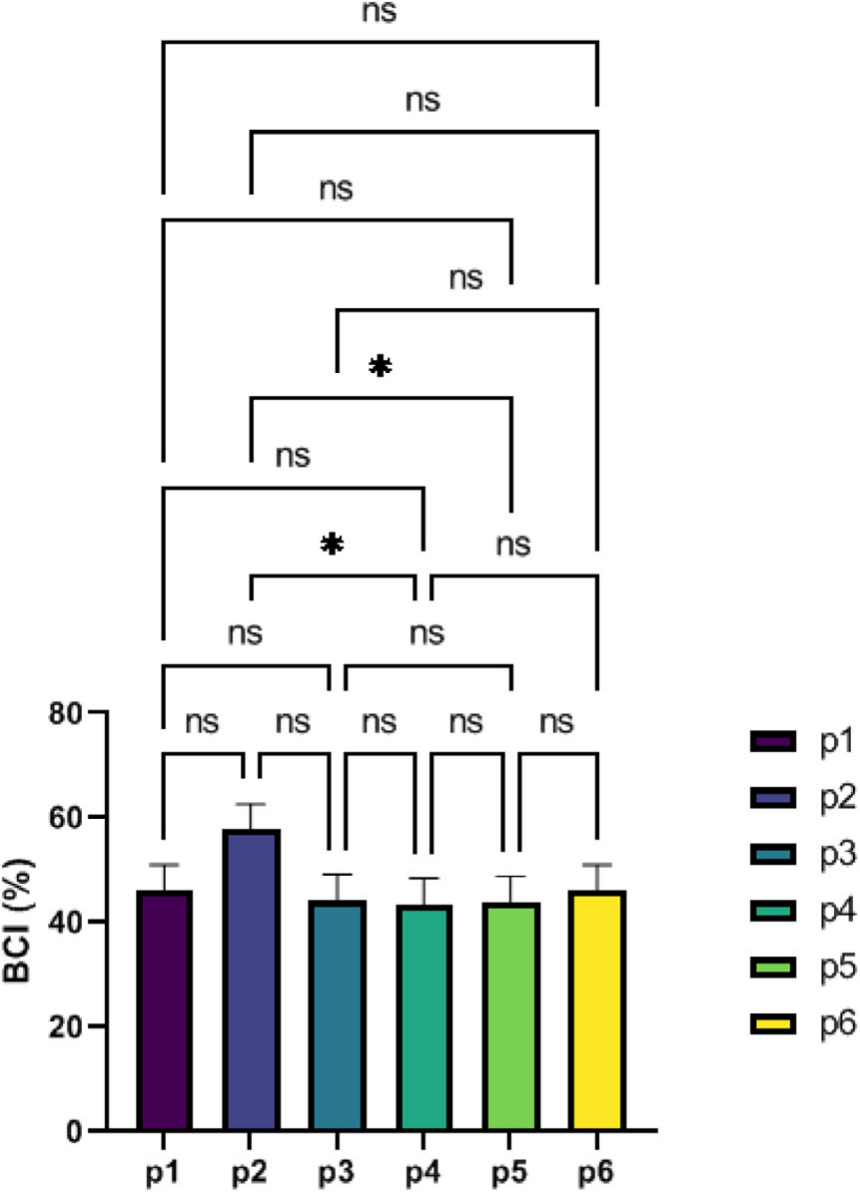
Blood coagulation index (BCI) of eggshell membrane. Values represent the mean ± SD, *n *= 3. Significance levels are expressed as ****p < .0001, ***p < .001, **p < .01, and *p < .05.

The whole blood coagulation index (BCI) primarily represents the effect of dressings on coagulation. With a lower BCI, biomaterials prevent blood clotting. The best protocol was P4, whose BCI value was 43.27 ± 5% (*n* = 3). However no significant difference between the other groups (Figure 8). The findings showed that the ESM attained a satisfactory level of coagulation.

### 3.8 In vitro degradation study

**FIGURE 9:**
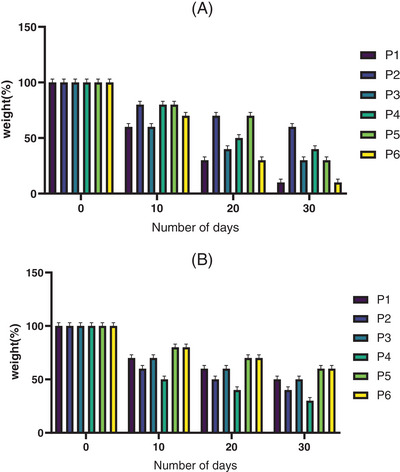
Biodegradation of eggshell membrane. (A) Trypsin and (B) PBS in different time points. Values represent the mean ± SD, *n* = 3.

Biodegradation of ESM was performed in PBS and trypsin at standard settings (pH 7.4; 37°C) in the laboratory. The retained percentage weight is displayed in Figure 9. ESM degradation in P4 was faster in PBS than in the other protocols. The degradation speed of the material should correspond with the regeneration rate of the tissue. The slow rate of material degradation hurts the replacement of the growing tissue. In the presence of trypsin, however, the degradation rate was accelerated. Moreover, the percentages of degradation in all groups were very close together. So the ESM is a degradable material.

### 3.9 ESM's microenvironmental pH

A favorable environment for wound repair can be created by adjusting several factors like carbon dioxide or oxygen levels, temperature, and PH. A proper microenvironmental pH may indirectly modify the interior environment of the injury by stimulating fibroblast proliferation.^40^ As a result, a suitable material should have a pH between 4.0 and 6.8, comparable to that of natural, healthy skin. Table 1 demonstrated that all the ESMs had a somewhat acidic pH (between 5.08 and 5.37), which is consistent with pH levels reported by previous researchers for commercial wound care products and there is no significant change in results over time.

**TABLE 1:** pH values of different various treated groups.

Time point	P1	P2	P3	P4	P5	P6
6 h	5.32 ± 0.08%	5.14 ± 0.09%	5.17 ± 0.12%	5.37 ± 0.14%	5.22 ± 0.08%	5.08 ± 0.13%
24 h	5.33 ± 0.06%	5.11 ± 0.12%	5.15 ± 0.16%	5.35 ± 0.23%	5.24 ± 0.19%	5.08 ± 0.04%
48 h	5.29 ± 0.11%	5.12 ± 0.03%	5.15 ± 0.09%	5.38 ± 0.12%	5.19 ± 0.26%	5.09 ± 0.09%

*Note*: Values indicate the average ± standard, *n* = 3.

## 4 DISCUSSION

Tissue engineering disciplines recognize collagen‐based biomaterials as a crucial component due to their ability to provide an ECM basis for tissue regeneration. The presence of collagen (type I, V, and X) in chicken ESM makes it a perfect substitute for ECM in the regeneration of tissue.^41^

According to this finding, we presented the identification and preparation of the neutral ESM as a biomaterial for skincare products. ESM is a popular unique biomaterial made up of polysaccharides, antimicrobial proteins, glycoproteins, and collagen.^42^ These components are present in significant concentrations in ESMs compared to other commercial sources and may require less processing to extract them.^43^ The organic material ESM has been proven to boost the activity of cells and collagen formation. Moreover, ESM inhibits skin aging as well as lowers UV light and inflammation‐related damage.^43^ ESM has a rich history of use as a wound‐healing product because it increases collagen deposition, which results in thicker granulation tissue and accelerates wound healing.^44^ Moisturizers, wound healing, skin expansion, and antiwrinkle agents are only some of the applications proposed for ESM.^45^ Also, many studies have examined the ESM as a different biomaterial. Additionally, in vitro and in vivo research ESM has been utilized. Clinical studies confirmed its efficacy in alleviating pain, reducing joint stiffness, and resolving connective tissue problems. Nevertheless, we anticipate that there will be more ESM commercial products soon due to their great potential. The best fabrication technique is required since even little changes to individual components might result in outputs with wildly varied chemical and mechanical properties.^26^

Finding a suitable extraction technique that maintains the ESM composition and can be applied on a large commercial scale is necessary, given the expanding use of ESM biomaterial. So in this study, a comparison was made between acid solutions often used to separate ESMs, revealing that the dissolution with HCl was the only approach that had blood compatibility, biodegradation, and cell viability.

The FTIR demonstrated that dissolution with HCl, compared to the other methods of ESM separation, could also lead to a shift in the chemical structure, which could affect its qualities as a biomaterial. Acid disintegration of the mineral in eggshells is a standard method utilized by numerous researchers that employ the ESM as a biocompatible material.^38^

The porous structure in materials is a suitable environment for cellular processes, including adhesion and proliferation.^46^ It is recognized that the morphology of materials influences cell adhesion. Although the meshwork shape of the surface of ESM could provide a physical framework for cell support, it is also conducive to cell adhesion and growth.^47^ The SEM pictures were utilized to analyze the porosity structure of the created ESM. It was somewhere between 416.5 nm to 6.952 µm, which is compatible with cellular processes.

At the initial stage of determining a material's biocompatibility, cytotoxicity experiments using cell culture‐based techniques are carried out.^7^ The present investigation demonstrates that the ESMs are biocompatible and have no harmful effects on 3T3 cells. The MTT test results indicated that ESMs could boost cell viability. Besides, P4 had the highest cell viability.

The utilization of biomimetic scaffolds such as ESM can enhance wound healing, prevent pathogen‐mediated infection, and decrease scarring.^48^ The wound healing assay can measure fundamental cell migration factors like speed, persistence, and polarity.^49^ The results of the wound healing tests revealed that it significantly impacts the in vitro migration rate of 3T3 cells; it will be beneficial for re‐epithelization during wound healing. Furthermore, our investigation confirms that P4 had a higher migration rate than other protocols and significantly improved wound area in vitro, which can be due to its greater cell compatibility and reproductive potential.

The ASTM F756 hemolytic index says that a material is hemolytic when the percentage of hemolysis is more than 5%, slightly hemolytic when the rate of hemolysis is between 2 and 5%, and not hemolytic when the ratio of hemolysis is <2%.^50^ As a result, all protocols are hemolytic, suggesting that they are to some extent compatible with blood. However, because the ESM is not directly exposed to blood, blood problems will not occur after ESM transplantation on the wound area.

The blood clotting index (BCI) calculates the absorption spectrum of hemoglobin freed from nonattached RBCs to assess the formation of stable clots.^51^ The results indicated that the ESM had acquired acceptable coagulation. Based on the BCI results, P4 is better than other protocols, which can be due to its final washing with NaOH, because based on past studies, sodium hydroxide can lead to the formation of clots and increase the coagulation speed.^52^

The process of biodegradation of biological materials is a vital feature in wound healing because it leads to the release of effective factors in the structure of the scaffold and biomaterial to the repair site, and on the other hand, the speed of biodegradation must be consistent and proportional with tissue regeneration.^53^ As time progressed, the percentage of destruction increased in all groups. Therefore, our research confirms that the ESM is a degradable material and according to the timing of the stages of wound healing, especially the homeostasis phase, it can be more effective due to blood compatibility.

Furthermore, microenvironmental pH in all groups revealed that the ESM created in this work could function as an acidic condition to inhibit bacterial invasion and indirectly promote fibroblast proliferation throughout the wound‐healing process.^54^

One of the most important limitations of this study is the lack of examination of the wound healing rate in the animal model, which provides a better and deeper research ability by examining the histology of ESM function. It is suggested that in future studies, the obtained protocol should be used for further investigations in all types of burn, full‐thickness, and diabetic wounds.

## 5 CONCLUSIONS

This paper described six valuable methods for extracting the entire membrane from the eggshell. Thus, P4 is superior to other protocols separating the ESM for skin regeneration beacuase more hemo and cell‐compatible. The ESM's defined qualities were determined using biocompatibility, biodegradability, and physicomechanical characterization. To evaluate the ESM's efficacy in an animal model, additional in vivo testing will be required. On the whole, the report shows that the ESMs can be used in tissue regeneration to speed up healing after injuries or skin trauma. Currently, there are no commercial products based on ESM designed for specific applications, though ESM supplements are available for purchase online. However, since P4 has been verified via physical, chemical, and biological analysis and is suitable for use on a large industrial scale, we anticipate that ESM commercial skincare products will increase soon.

## CONFLICT OF INTEREST STATEMENT

The authors declare no conflicts of interest or personal relationships that could have appeared to influence the work reported in this paper.

## Data Availability

Data will be made available on request.

## References

[1] Eyerich S , Eyerich K , Traidl‐Hoffmann C , Biedermann T . Cutaneous barriers and skin immunity: differentiating a connected network. Trends Immunol. 2018;39(4):315‐327. 29551468 10.1016/j.it.2018.02.004

[2] Eming SA , Martin P , Tomic‐Canic M . Wound repair and regeneration: mechanisms, signaling, and translation. Sci Transl Med. 2014;6(265):265sr6. 25473038 10.1126/scitranslmed.3009337 PMC4973620

[3] Guest JF , Ayoub N , McIlwraith T , et al. Health economic burden that wounds impose on the National Health Service in the UK. BMJ Open. 2015;5(12):e009283. 10.1136/bmjopen-2015-009283 PMC4679939 26644123

[4] Sen CK . Human wounds and its burden: an updated compendium of estimates. Adv Wound Care. 2019;8:39‐48. 10.1089/wound.2019.0946 PMC6389759 30809421

[5] Saghazadeh S , Rinoldi C , Schot M , et al. Drug delivery systems and materials for wound healing applications. Adv Drug Deliv Rev. 2018;127:138‐166. 29626550 10.1016/j.addr.2018.04.008 PMC6003879

[6] Silva SS , Mano JF , Reis RL . JBAfBD. Soft constructs for skin tissue engineering. 2012:537‐557, DOI:10.1002/9783527652273

[7] Bernard M , Jubeli E , Pungente MD , Yagoubi N . Biocompatibility of polymer‐based biomaterials and medical devices – regulations, in vitro screening and risk‐management. Biomater Sci. 2018;6(8):2025‐2053. 29968869 10.1039/c8bm00518d

[8] Shi Y , Zhou K , Li D , Guyonnet V , Hincke MT , Mine Y . Avian eggshell membrane as a novel biomaterial: a review. Foods. 2021;10(9):2178. 34574286 10.3390/foods10092178 PMC8466381

[9] Karan SK , Maiti S , Paria S , et al. A new insight towards eggshell membrane as high energy conversion efficient bio‐piezoelectric energy harvester. Mater Today Energy. 2018;9:114‐125.

[10] Jia J , Duan YY , Yu J , Lu JW . The Japanese Society for Biomaterials,, Biomaterials TASf, Biomaterials tKSf. Preparation and immobilization of soluble eggshell membrane protein on the electrospun nanofibers to enhance cell adhesion and growth. J Biomed Mater Res A. 2008;86(2):364‐373. 17969029 10.1002/jbm.a.31606

[11] Kulshreshtha G , Ahmed TA , Wu L , Diep T , Hincke MT . A novel eco‐friendly green approach to produce particalized eggshell membrane (PEM) for skin health applications. Biomater Sci. 2020;8(19):5346‐5361. 32857070 10.1039/d0bm01110j

[12] Torres FG , Troncoso OP , Piaggio F , Hijar A . Structure–property relationships of a biopolymer network: The eggshell membrane. Acta Biomater. 2010;6(9):3687‐3693. 20227532 10.1016/j.actbio.2010.03.014

[13] Sheish SG , Emadi R , Ahmadian M , Sadeghzade S , Tavangarian F . Fabrication and characterization of polyvinylpyrrolidone‐eggshell membrane‐reduced graphene oxide nanofibers for tissue engineering applications. Polymers (Basel). 2021;13(6):913. 33809630 10.3390/polym13060913 PMC8002296

[14] Liu M , Liu T , Zhang X , et al. Fabrication of KR‐12 peptide‐containing hyaluronic acid immobilized fibrous eggshell membrane effectively kills multi‐drug‐resistant bacteria, promotes angiogenesis and accelerates re‐epithelialization. Int J Nanomedicine. 2019;14:3345. 31190796 10.2147/IJN.S199618 PMC6516050

[15] Torres‐Mansilla A , Delgado‐Mejía E . Influence of separation techniques with acid solutions on the composition of eggshell membrane. Int J Poult Sci. 2017;16:451‐456.

[16] Yoo J , Park K , Yoo Y , Kim J , Yang H , Shin YJ . Effects of egg shell membrane hydrolysates on anti‐inflammatory, anti‐wrinkle, anti‐microbial activity and moisture‐protection. Korean J Food Sci Anim Resour. 2014;34(1):26‐32. 26760742 10.5851/kosfa.2014.34.1.26 PMC4597828

[17] Ruff KJ , DeVore DP . Reduction of pro‐inflammatory cytokines in rats following 7‐day oral supplementation with a proprietary eggshell membrane‐derived product. Mod Res Inflamm. 2014;2014:19‐25.

[18] Jun HJ , Oh K‐H , Yoo J , et al. A new patch material for tympanic membrane perforation by trauma: the membrane of a hen egg shell. Acta Otolaryngol. 2014;134(3):250‐254. 24320021 10.3109/00016489.2013.857784

[19] Guarderas F , Leavell Y , Sengupta T , Zhukova M , Megraw TL . Assessment of chicken‐egg membrane as a dressing for wound healing. Adv Skin Wound Care. 2016;29(3):131‐134. 26866869 10.1097/01.ASW.0000480359.58866.e9

[20] Taylor KR , Gallo RL . Glycosaminoglycans and their proteoglycans: host‐associated molecular patterns for initiation and modulation of inflammation. FASEB J. 2006;20(1):9‐22. 16394262 10.1096/fj.05-4682rev

[21] Mensah RA , Jo SB , Kim H , et al. The eggshell membrane: a potential biomaterial for corneal wound healing. J Biomater Appl. 2021;36(5):912‐929. 34139891 10.1177/08853282211024040 PMC8606947

[22] Vuong TT , Rønning SB , Ahmed TA , et al. Processed eggshell membrane powder regulates cellular functions and increase MMP‐activity important in early wound healing processes. PLoS One. 2018;13(8):e0201975. 30080894 10.1371/journal.pone.0201975 PMC6078314

[23] Choi J , Lee J , Shin ME , Been S , Lee DH , Khang GJP . Eggshell membrane/gellan gum composite hydrogels with increased degradability, biocompatibility, and anti‐swelling properties for effective regeneration of retinal pigment epithelium. Polymers (Basel). 2020;12(12):2941. 33317040 10.3390/polym12122941 PMC7764595

[24] Kessi E , Arias JL . Using natural waste material as a matrix for the immobilization of enzymes: chicken eggshell membrane powder for β‐galactosidase immobilization. Appl Biochem Biotechnol. 2019;187(1):101‐115. 29948996 10.1007/s12010-018-2805-4

[25] Santos KO , Barbosa RC , da Silva Buriti J , et al. Thermal, chemical, biological and mechanical properties of chitosan films with powder of eggshell membrane for biomedical applications. J Therm Anal Calorim. 2019;136(2):725‐735.

[26] Park S , Choi KS , Lee D , et al. Eggshell membrane: review and impact on engineering. Biosyst Eng. 2016;151:446‐463.

[27] Jung O , Smeets R , Porchetta D , et al. Optimized in vitro procedure for assessing the cytocompatibility of magnesium‐based biomaterials. Acta Biomaterialia. 2015;23:354‐363. 26073090 10.1016/j.actbio.2015.06.005

[28] Mansur H , Oréfice R , Vasconcelos W , Lobato Z , Machado L . Biomaterial with chemically engineered surface for protein immobilization. J Mater Sci: Mater Med. 2005;16(4):333‐340. 15803278 10.1007/s10856-005-0632-y

[29] Kumar P , Nagarajan A , Uchil PD . Analysis of cell viability by the MTT assay. Cold Spring Harb Protoc. 2018;2018(6):pdbprot095505. 10.1101/pdb.prot095505 29858338

[30] Cory G . Scratch‐wound assay. In: Cell Migration: Developmental Methods *and* Protocols. Humana Press. 2011:25‐30. 10.1007/978-1-61779-207-6_2 21748666

[31] Milne SD , Seoudi I , Al Hamad H , et al. A wearable wound moisture sensor as an indicator for wound dressing change: an observational study of wound moisture and status. Int Wound J. 2016;13(6):1309‐1314. 26561281 10.1111/iwj.12521 PMC7950073

[32] Dawids S . Test Procedures for the Blood Compatibility of Biomaterials. Springer Science & Business Media; 2012.

[33] Liu X , Niu Y , Chen KC , Chen S . Rapid hemostatic and mild polyurethane‐urea foam wound dressing for promoting wound healing. Mater Sci Eng C. 2017;71:289‐297. 10.1016/j.msec.2016.10.019 27987710

[34] Kolarijani NR , Mirzaii M , Zamani S , et al. Assessment of the ability of Pseudomonas aeruginosa and Staphylococcus aureus to create biofilms during wound healing in a rat model treated with carboxymethyl cellulose/carboxymethyl chitosan hydrogel containing EDTA. Int Wound J. 2024;21(5):e14878. 38682897 10.1111/iwj.14878 PMC11057379

[35] Percival SL , McCarty S , Hunt JA , Woods EJ . The effects of pH on wound healing, biofilms, and antimicrobial efficacy. Wound Repair Regen. 2014;22(2):174‐186. 24611980 10.1111/wrr.12125

[36] Choi HJ , Kim YM , Suh J‐Y , Han JY . Beneficial effect on rapid skin wound healing through carboxylic acid‐treated chicken eggshell membrane. Mater Sci Eng C. 2021;128:112350. 10.1016/j.msec.2021.112350 34474899

[37] Barth A . Infrared spectroscopy of proteins. Biochim Biophys Acta Bioenerg. 2007;1767(9):1073‐1101. 10.1016/j.bbabio.2007.06.004 17692815

[38] Baláž M , Zorkovská A , Fabián M , Girman V , Briančin J . Eggshell biomaterial: characterization of nanophase and polymorphs after mechanical activation. Adv Powder Technol. 2015;26(6):1597‐1608.

[39] Cheng H , Li C , Jiang Y , et al. Facile preparation of polysaccharide‐based sponges and their potential application in wound dressing. J Mater Chem B. 2018;6(4):634‐640. 32254492 10.1039/c7tb03000b

[40] Castano O , Pérez‐Amodio S , Navarro‐Requena C , Mateos‐Timoneda MÁ , Engel E . Instructive microenvironments in skin wound healing: biomaterials as signal releasing platforms. Adv Drug Deliv Rev. 2018;129:95‐117. 29627369 10.1016/j.addr.2018.03.012

[41] Shen X , Nagai N , Murata M , Nishimura D , Sugi M , Munekata M . Development of salmon milt DNA/salmon collagen composite for wound dressing. J Mater Sci: Mater Med. 2008;19:3473‐3479. 18592347 10.1007/s10856-008-3512-4

[42] Liu Z , Zhang F , Li L , Li G , He W , Linhardt RJ . Compositional analysis and structural elucidation of glycosaminoglycans in chicken eggs. Glycoconj J. 2014;31(8):593‐602. 25218438 10.1007/s10719-014-9557-3 PMC4229509

[43] Nys Y , Gautron J , Garcia‐Ruiz JM , Hincke MT . Avian eggshell mineralization: biochemical and functional characterization of matrix proteins. Comptes Rendus Palevol. 2004;3(6‐7):549‐562.

[44] Ahmed TA , Suso H‐P , Maqbool A , Hincke MT . Processed eggshell membrane powder: bioinspiration for an innovative wound healing product. Mater Sci Eng C. 2019;95:192‐203. 10.1016/j.msec.2018.10.054 30573242

[45] Park K‐M , Yoo J‐H , Shin Y‐J . Effects of egg shell membrane hydrolysates on skin whitening,wound healing, and UV‐protection. Food Science of Animal Resources. 2012;32(3):308‐315.

[46] Been S , Choi J , Cho H , et al. Preparation and characterization of a soluble eggshell membrane/agarose composite scaffold with possible applications in cartilage regeneration. J Tissue Eng Regen Med. 2021;15(4):375‐387. 33533202 10.1002/term.3178

[47] Yi F , Guo Z‐X , Zhang L‐X , Yu J , Li QJB . Soluble eggshell membrane protein: preparation, characterization and biocompatibility. Biomaterials. 2004;25(19):4591‐4599. 15120504 10.1016/j.biomaterials.2003.11.052

[48] Leite VC , Pereira Filho WW , de Oliveira Junior EN , Fabrino D . Cell viability evaluation of vero cells viability cultured on different chitosan films: development of functional biodressings possibilities. Int JAdv Med Biotechnol‐IJAMB. 2020;3(1):34‐39.

[49] Cory G . Scratch‐wound assay. In: Cell Migration. Springer; 2011:25‐30. 10.1007/978-1-61779-207-6_2 21748666

[50] Zamani S , Rezaei Kolarijani N , Naeiji M , et al. Development of carboxymethyl cellulose/gelatin hydrogel loaded with Omega‐3 for skin regeneration. J Biomater Appl. 2024;39(4):377-395. 39049504 10.1177/08853282241265769

[51] Xie X , Li D , Chen Y , et al. Conjugate electrospun 3D gelatin nanofiber sponge for rapid hemostasis. Adv Healthc Mater. 2021;10(20):2100918. 10.1002/adhm.202100918 34235873

[52] Lee C . Enhancing photothermal therapy via blood clot induced by needle‐type sodium hydroxide‐loaded starch implant. Colloids Interface Sci Commun. 2024;59:100773.

[53] Singh A , Ward OP . Biodegradation and Bioremediation. Springer Science & Business Media; 2004.

[54] Wang M , Yuan J , Huang X , et al. Grafting of carboxybetaine brush onto cellulose membranes via surface‐initiated ARGET‐ATRP for improving blood compatibility. Colloids Surf B Biointerfaces. 2013;103:52‐58. 23201719 10.1016/j.colsurfb.2012.10.025

